# Endoscopic submucosal dissection for colorectal neoplasms: Risk factors for local recurrence and long‐term surveillance

**DOI:** 10.1002/deo2.269

**Published:** 2023-07-02

**Authors:** Taishi Okumura, Takemasa Hayashi, Shin‐ei Kudo, Kenichi Mochizuki, Masahiro Abe, Tatsuya Sakurai, Yuta Kouyama, Yushi Ogawa, Yasuharu Maeda, Naoya Toyoshima, Masashi Misawa, Toyoki Kudo, Kunihiko Wakamura, Toshiyuki Baba, Fumio Ishida, Hideyuki Miyachi

**Affiliations:** ^1^ Digestive Disease Center Showa University Northern Yokohama Hospital Kanagawa Japan

**Keywords:** endoscopic submucosal dissection, local recurrence, non‐R0 resection, risk factors, surveillance colonoscopy

## Abstract

**Objectives:**

Endoscopic submucosal dissection (ESD) is an effective procedure for the en bloc resection of colorectal neoplasms. However, risk factors for local recurrence after ESD have not been identified. This study aimed to evaluate such risk factors after ESD for colorectal neoplasms.

**Methods:**

This retrospective study included 1344 patients with 1539 consecutive colorectal lesions who underwent ESD between September 2003 and December 2019. We investigated various factors associated with local recurrence in these patients. The main outcomes were the incidence of local recurrence and its relationship with clinicopathological factors during long‐term surveillance.

**Results:**

The en bloc resection rate was 98.6%, the R0 resection rate was 97.2%, and the histologically complete resection rate was 92.7%. Local recurrence was observed in 7/1344 (0.5%) patients and the median follow‐up period was 72 months (range 4–195 months). The incidence of local recurrence was significantly higher in lesions ≥40 mm in diameter (hazard ratio [HR] 15.68 [1.88–130.5]; *p* = 0.011), piecemeal resection (HR 48.42 [10.7–218.7]; *p* < 0.001), non‐R0 resection (HR 41.05 [9.025–186.7]; *p* < 0.001), histologically incomplete resection (HR 16.23 [3.627–72.63]; *p*<0.001), and severe fibrosis (F2; HR 9.523 [1.14–79.3]; *p* = 0.037).

**Conclusions:**

Five risk factors for local recurrence after ESD were identified. Patients with such factors should undergo careful surveillance colonoscopy.

## INTRODUCTION

Endoscopic submucosal dissection (ESD) is an effective procedure for the en‐bloc resection of large colorectal neoplasms.^1^, ^2^, ^3^, ^4^, ^5^ Compared to endoscopic mucosal resection, it is technically more difficult, requires considerable experience, and has a greater risk of adverse events such as perforation.^3^, ^6^, ^7^, ^8^ Elucidating the factors affecting ESD technical difficulty,^9^, ^10^, ^11^, ^12^, ^13^ the improvement of tools and devices,^4^ and establishing a training system^14^ have increased its safety.

Several studies have reported remarkable outcomes after ESD for colorectal neoplasms^3^, ^15^, ^16^, ^17^, ^18^, ^19^, ^20^; some suggest that piecemeal resection is the only risk factor associated with local recurrence after ESD.^17^, ^18^, ^19^, ^21^, ^22^ However, their median follow‐up periods were only 2 years. Few reports of local recurrence after en‐bloc resection are available,^23^, ^24^ and long‐term data on numerous cases are needed to confirm colorectal ESD as a technique with more favorable long‐term clinical outcomes and very low recurrence rates. In this single‐center retrospective cohort study, we surveyed patients undergoing ESD to determine the long‐term local recurrence rates of colorectal ESD and to clarify the clinicopathological characteristics of local recurrent lesions after ESD.

## METHODS

### Inclusion criteria

Between September 2003 and December 2019, 31,615 colorectal lesions (excluding stage >T2 carcinomas) were resected endoscopically or surgically at Showa University Northern Yokohama Hospital, Yokohama, Japan, and 1504 patients underwent ESD for 1724 lesions. Among these, 1344 (1539 lesions) were followed up on at least once (for local recurrence surveillance) and enrolled in this study (follow‐up rate: 89.3%). All patients with a successful initial ESD who had undergone at least the first surveillance colonoscopy were included. If a single patient had multiple lesions, they were counted by the number of lesions. However, the recurrent lesions were excluded due to the risk of skip lesions possibly going undetected during initial ESD.

### Indication for ESD

The indications for colorectal ESD were defined according to the Japan Gastroenterological Endoscopy Society colorectal ESD/endoscopic mucosal resection guidelines as follows: (1) colorectal neoplasms >20 mm in diameter, with a preoperative diagnosis through narrow‐band imaging magnification and chromoendoscopic magnification of the possible mucosal tumor or slightly invasive submucosal cancer, for which en bloc resection would be difficult—including a laterally spreading tumor, non‐granular type (particularly the pseudo‐depressed type), large depressed type, and large protruded type suspected to be a carcinoma; (2) mucosal lesions or early‐stage cancers with fibrosis caused by previous treatment, biopsy, or colonic wall peristalsis; and (3) sporadic and localized lesions in chronic intestinal inflammation such as ulcerative colitis.^25^

### Procedure and devices of ESD

ESD was performed by seven endoscopists specialized in endoscopic colorectal treatment (procedure and devices of ESD described in previous reports).^26^, ^27^, ^28^ The following endoscopes were used: a water‐jet system (GIF‐Q260J; Olympus, Tokyo, Japan [September 2003–September 2008]) gastroscope and two water‐jet systems (PCF‐Q260JI; Olympus [October 2008–February 2018] and PCF‐H290TI; Olympus [from March 2018]) colonoscopes. A triangle‐tip knife (KD‐630L; Olympus [September 2003–September 2008]) and a flush knife (DK2618LN; Fujifilm Medical, Tokyo, Japan [from October 2008]) were used as endo‐knives. A transparent hood (D‐201‐11804; Olympus) was attached to the tip of the endoscope to enhance field visualization and ensure stable dissection. From September 2003 to March 2008, the injected agent was a 1% hyaluronic acid solution (Suvenyl; Chugai Pharmaceutical, Tokyo, Japan) mixed with a 10% glycerin, 5% fructose, and 0.9% saline solution (Glyceol; Chugai Pharmaceutical). From April 2008, a 0.4% hyaluronic acid solution (Mucoup; Johnson & Johnson K.K., Tokyo, Japan) was used.^29^, ^30^ The electrosurgical units used were the ICC 200 (Erbe Elektromedizin, Tübingen, Germany [September 2003–August 2018]) and the VIO3 (Erbe Elektromedizin, Tübingen, Germany [from September 2018]).

### Histopathological assessment

All resected lesions were fixed in a 10% buffered formalin solution for 24–48 h and observed with a focus on the pit pattern using a stereomicroscope. They were incised at the point where the deepest area of invasion could be exposed on the surface of the cut end. The other specimens were cut into parallel 2–3‐mm‐thick sections. En bloc resection was defined as the one‐piece resection of an entire lesion, as observed endoscopically. The following resections were defined: complete (R0) en bloc resections with tumor‐free lateral and vertical margins; R1 en bloc or piecemeal resections with positivity for tumor cells on the horizontal resection margin or vertical resection margin, respectively; and RX resections with a lateral resection margin that could not be assessed in piecemeal resection. Histologically complete resection, based on the Japanese Society for Cancer of the Colon and Rectum (JSCCR) guidelines, was confirmed when the pathological findings showed R0 or RX resection with none of the following features: well/moderately differentiated or papillary carcinoma, no vascular invasion, submucosal invasion depth <1000 μm, and budding grade 0/1.^31^ The histologies of the lesions were classified as adenoma, Tis (intramucosal cancer), and T1 (submucosal invasive cancer).

### Surveillance after ESD

Surveillance colonoscopy was mostly performed 6 months after ESD; however, in some cases such as non‐R0 resection, it was performed within 6 months. A local recurrent lesion was defined as one whose center was detected within the ESD scar upon surveillance colonoscopy. Patients with histologically complete resection were followed‐up on using colonoscopy 1, 3, and 5 years after ESD, with abdominal computed tomography (CT) scans as required. Magnifying colonoscopy with clinical classification was routinely used in the surveillance colonoscopy. Additional surgery was strongly recommended for patients with histologically incomplete resection, except for those with pathology specimens positive only in the lateral resection margin. If these patients refused additional surgery, they were followed up on using CT scans, abdominal ultrasonography, and the measurement of serum tumor marker levels (CA19‐9 and CEA) every 6 months for at least 5 years, in addition to an annual colonoscopy.

### Outcome measures

Various factors associated with local recurrence after ESD were retrospectively analyzed, including age, sex, lesion location and size, morphological type, resection status, histopathology, submucosal layer fibrosis, and adverse events. The lesion locations were classified into three categories: right colon, including the cecum, ascending, and transverse colon; left colon, including the descending and sigmoid colon; and rectum. Morphological types were classified as non‐polypoid‐containing depressed, laterally spreading tumor granular (LST‐G), non‐granular (LST‐NG), or polypoid‐containing protruded and pedunculated types. Endoscopically, the degree of submucosal fibrosis was classified as reported previously: no fibrosis (F0), mild fibrosis (F1), and severe fibrosis (F2).^5^ Adverse events were evaluated for perforation (defect of the muscular layer at the ESD ulcer) and delayed bleeding (intermittent hematochezia occurring after colonoscopy withdrawal and requiring endoscopic hemostasis).

### Ethical considerations

This study was performed in accordance with the Declaration of Helsinki. All patients were informed of the risks and benefits of ESD, and each provided written informed consent for the procedure. The ethics committee of our hospital approved the study protocol (approval number: 20H110). This study was registered with the University Hospital Medical Network Clinical Trials Registry (UMIN000045465).

### Statistical analysis

Values are reported as means and standard deviations (SDs). We performed univariate analyses with Cox proportional hazard regression analysis to identify lesion, patient, and treatment factors associated with local recurrence. Hazard ratios (HRs) and 95% confidence intervals (95% CIs) were calculated for the total population and each treatment group. Furthermore, the local recurrence rate was calculated using the Kaplan–Meier method. Statistical significance was set at a *p*‐value < 0.05. All statistical analyses were performed using EZR (version 1.56; Saitama Medical Center, Jichi Medical University, Saitama, Japan), a graphical user interface for R software (The R Foundation for Statistical Computing, Vienna, Austria).

## RESULTS

### Outcomes related to ESD

**FIGURE 1:**
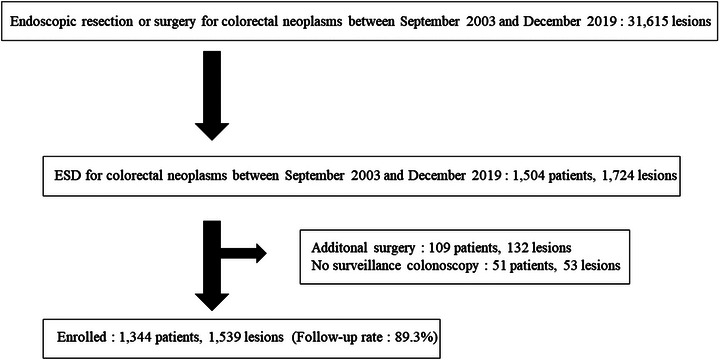
Flowchart of enrolled patients and lesions.

ESD was performed on 1724 colorectal lesions in 1504 patients. Of these, 160 patients (185 lesions) have not yet received their follow‐up endoscopic examination. In total, 109 patients (132 lesions) underwent surveillance colonoscopy for marking due to additional surgery for histologically incomplete resection; however, it was too early to estimate the presence of a recurrent lesion. Some of these patients had multiple lesions, including ESD lesions curatively resected, but within the range of surgical resection; however, as there was no residual lesion in the surgical specimen, these patients were not enrolled. The remaining 51 patients (53 lesions) did not receive surveillance colonoscopy for any reason. Finally, surveillance colonoscopy after ESD was conducted in 1539 colorectal lesions of 1344 patients (Figure 1).

The clinicopathological characteristics of enrolled patients and lesions are presented in Table 1. The mean ± SD age of the patients was 67.6 ± 10.9 years, the male‐to‐female ratio was 804:540, and the mean ± SD lesion size was 31.2 ± 11.2 mm. Additionally, the en bloc, R0, and histologically complete resection rates were 98.6% (1518/1539), 97.2% (1496/1539), and 92.7% (1427/1539), respectively. There were 444 (28.8%) adenomas, 898 (58.4%) Tis, and 197 (12.8%) T1 tumors. Furthermore, severe fibrosis (F2) of the submucosal layer under the lesion was observed in 29/1539 lesions (1.9%), while the rate of perforation was 2.8% (43/1539) and the rate of delayed bleeding was 1.4% (21/1539). All adverse events were successfully managed endoscopically.

**TABLE 1:** Clinicopathologic characteristics of the enrolled patients and lesions (median follow‐up period 72 months, range 4–195 months).

**Variables**	**Total (%)**
**Patients, *n***	1344
**Age (years)**:	
Average ± SD	67.6 ± 10.9
<60	276 (20.5)
≥60	1068 (79.5)
**Sex**:	
Male	804 (59.8)
Female	540 (40.2)
**Antithrombotic treatment**:	
+	174 (12.9)
−	1170 (87.1)
**Lesions, n**	1539
**Location of lesion**:	
Right colon	864 (56.1)
Left colon	335 (21.8)
Rectum	340 (22.1)
**Size of the lesion (mm)**:	
Average ± SD	31.2 ± 11.2
<40	1137 (73.9)
≥40	402 (26.1)
**Morphological type**:	
Non‐polypoid	1457 (94.7)
Polypoid	82 (5.3)
**Resection status**:	
En bloc	1518 (98.6)
Piecemeal	21 (1.4)
**Histology**:	
Adenoma	444 (28.8)
Tis	898 (58.4)
T1 (<1000 μm)	106 (6.9)
T1 (≥1000 μm)	91 (5.9)
**Endoscopic curability**:	
R0 resection	1496 (97.2)
Non‐R0 resection	43 (2.8)
**Histologically complete resection**:	
Complete	1427 (92.7)
Incomplete	112 (7.3)
**Fibrosis**:	
F0, F1	1510 (98.1)
F2	29 (1.9)
**Perforation**:	
+	43 (2.8)
−	1496 (97.2)
**Delayed bleeding**:	
+	21 (1.4)
−	1518 (98.6)

Abbreviations: SD, standard deviation; Tis, intramucosal cancer; T1, submucosal invasive cancer.

### Clinicopathological and treatment factors related to local recurrence

**FIGURE 2:**
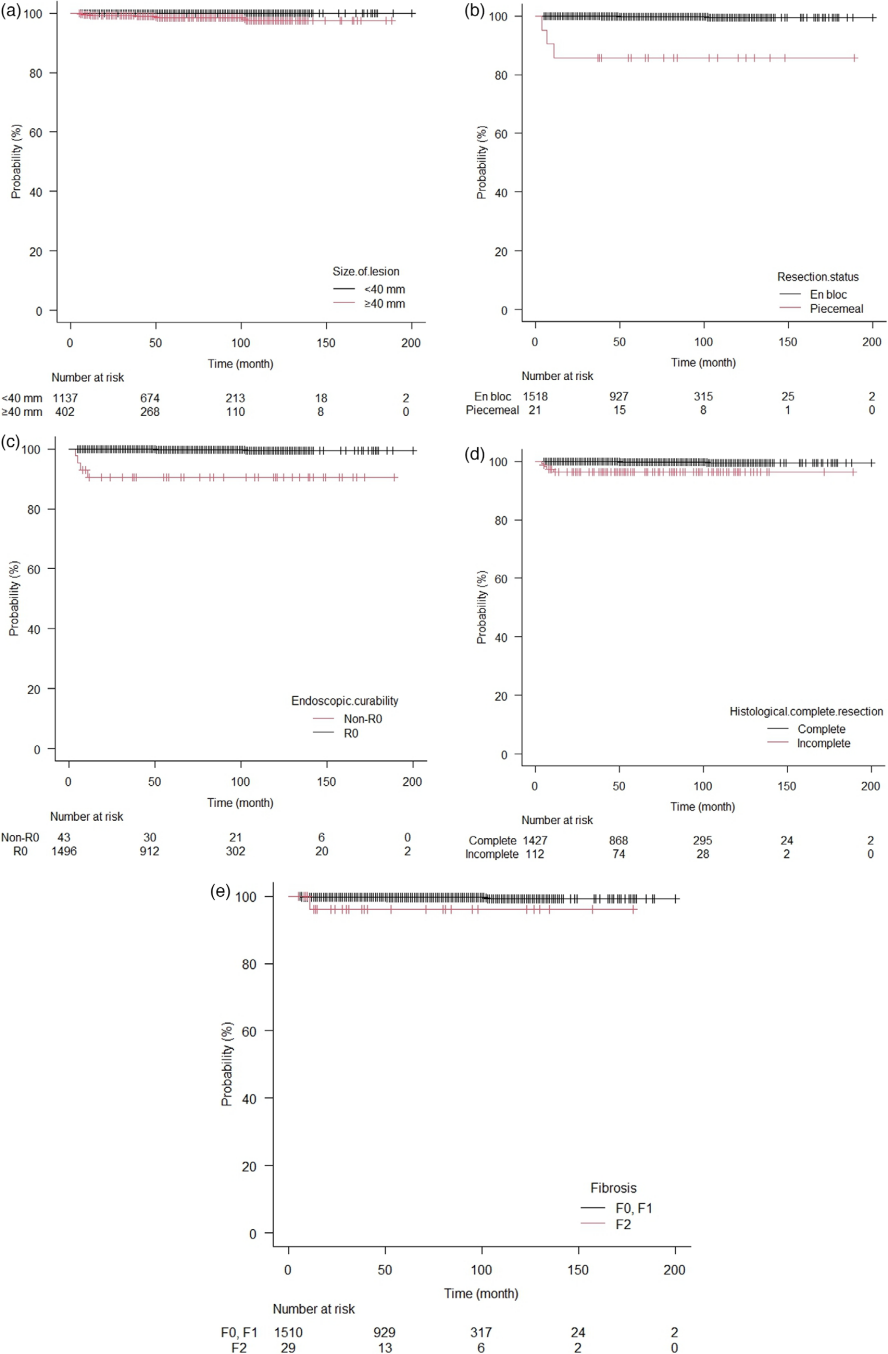
Kaplan–Meier curve comparing the local recurrence rate. (a) Size of the lesion; (b) Resection status; (c) Endoscopic curability; (d) Histologically complete resection; (e) Fibrosis.

Local recurrence was observed in 7/1344 (0.5%) patients and the median follow‐up period was 72 months (range, 4–195 months). The clinicopathological and treatment factors associated with local recurrence after ESD are shown in Table 2. We found that the incidence of local recurrence according to univariate analyses with Cox proportional hazard regression analysis was significantly higher in lesions ≥40 mm in diameter (HR, 15.68 [1.88–130.5]; *p* = 0.011), piecemeal resection (HR, 48.42 [10.7–218.7]; *p* < 0.001), non‐R0 resection (HR, 41.05 [9.025–186.7]; *p* < 0.001), histologically incomplete resection, and severe fibrosis (F2; HR, 9.523 [1.14–79.3]; *p* = 0.037). Moreover, the Kaplan–Meier curve (Figure 2) revealed that the local recurrence rate was significantly higher in lesions ≥40 mm in diameter (*p* < 0.001), piecemeal resection (*p* < 0.001), non‐R0 resection (*p* < 0.001), histologically incomplete resection (HR, 16.23 [3.627–72.63]; *p* < 0.001), and severe fibrosis (F2; *p* = 0.011).

**TABLE 2:** Univariate analysis of risk factors for local recurrence after endoscopic submucosal dissection (ESD).

		Univariate analysis		
Variables	Local recurrence	HR	95% CI	*p*‐value
**Age (years)**:				
<60	1/321	1		
≥60	6/1218	1.583	0.19–13.2	0.671
**Sex**:				
Male	6/922	3.909	0.47–32.5	0.207
Female	1/617	1		
**Location of lesion**:				
Right colon	2/864	1		
Left colon	3/335	3.841	0.64–30.0	0.141
Rectum	2/340	2.386	0.33–17.0	0.385
**Size of the lesion (mm)**:				
<40	0/1137	1		
≥40	7/402	15.68	1.88–130.5	0.011
**Morphological type**:				
Non‐polypoid	6/1457	1		
Polypoid	1/82	3.182	0.38–26.5	0.284
**Resection status**:				
En bloc	5/1518	1		
Piecemeal	2/21	48.42	10.7–218.7	<0.001
**Depth of invasion**:				
m	5/1342	1		
sm	2/197	2.616	0.51–13.5	0.251
**Endoscopic curability**:				
R0 resection	3/1496	1		
Non‐R0 resection	4/43	41.05	9.025–186.7	<0.001
**Histologically complete resection**:				
Complete	3/1427	1		
Incomplete	4/112	16.23	3.627–72.63	<0.001
**Fibrosis**:				
F0, F1	6/1510	1		
F2	1/29	9.523	1.14–79.3	0.037
**Perforation**:				
+	1/43	<0.001	0–Inf	0.9981
−	6/1496	1		
**Delayed bleeding**:				
+	0/21	<0.001	0–Inf	0.9981
−	7/1518	1		

ESD, endoscopic submucosal dissection; HR, hazard ratio; CI, confidence interval; Inf, infinity

### Additional treatment for local recurrent lesions

**FIGURE 3:**
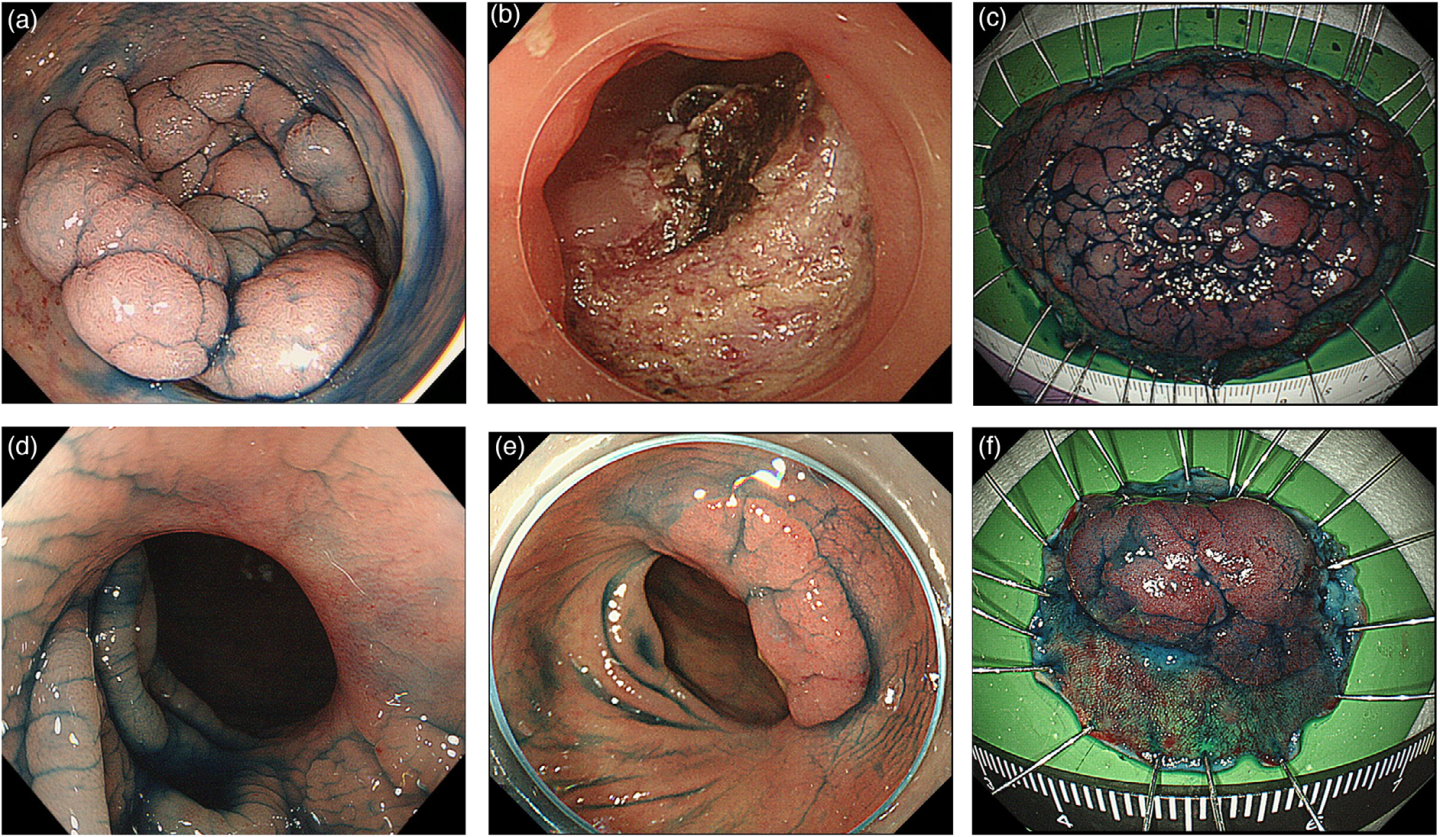
A 63‐year‐old man underwent histologically complete resection with endoscopic submucosal dissection (ESD) for transverse intramucosal cancer. (a) Laterally spreading tumor; (b) The ulcer bed after ESD: en bloc resection was performed using ESD; (c) Histopathological examination of the resected specimen showing intramucosal adenocarcinoma in adenomatous components (size: 90 × 50 mm), tumor‐free resection margins, and no evidence of lymphovascular invasion; (d) Surveillance colonoscopy after 4 months showed no recurrence of the ESD scar; (e) Surveillance colonoscopy after 38 months showed an elevated lesion on the ESD scar; (f) En‐bloc resection with ESD was performed for the recurrent lesion, which was pathologically diagnosed as an adenoma (HM0, VM0).

**FIGURE 4:**
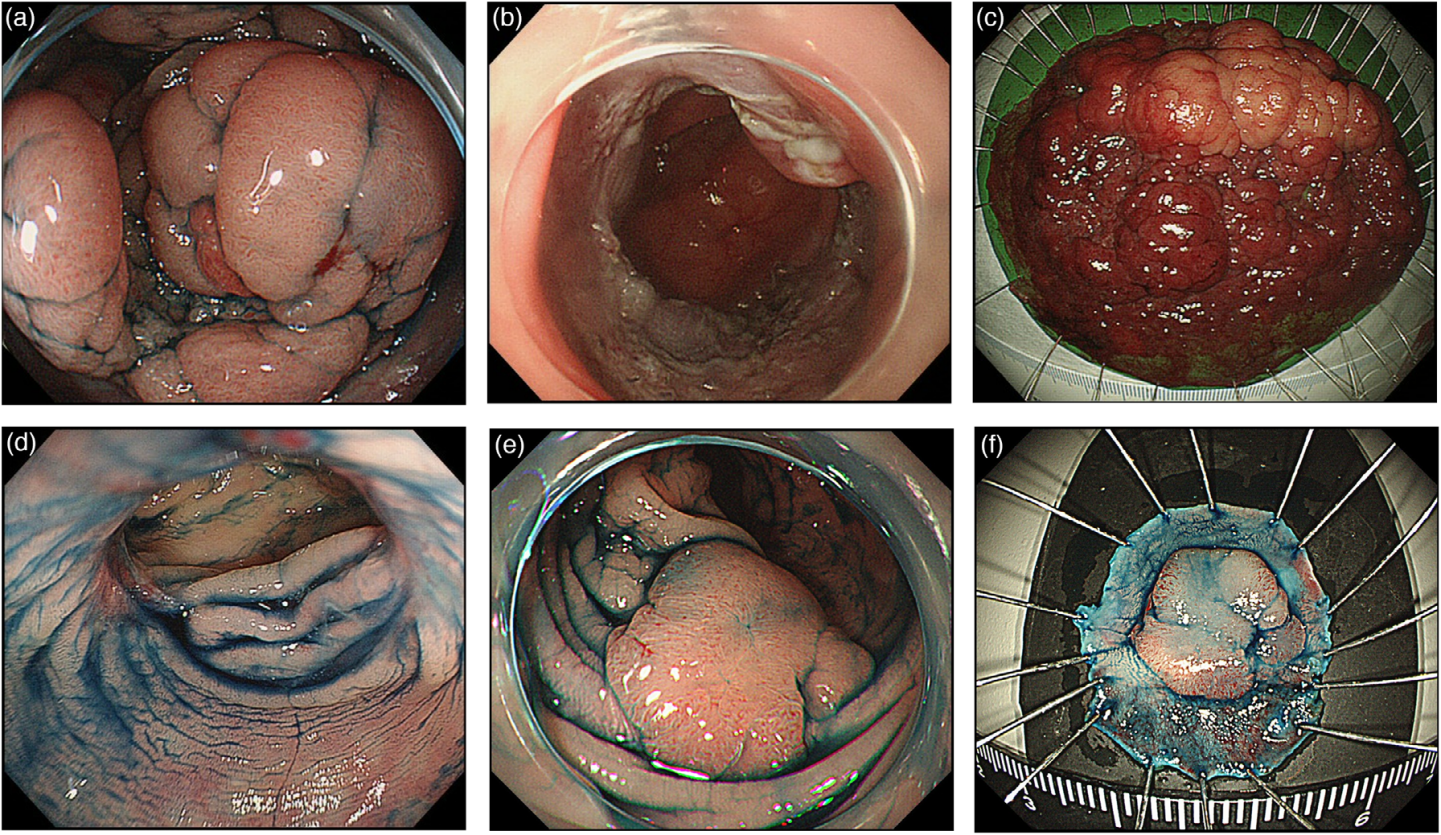
A 73‐year‐old man underwent histologically complete resection with endoscopic submucosal dissection (ESD) for sigmoid intramucosal cancer (a) Laterally spreading tumor; (b) The ulcer bed after ESD: en‐bloc resection was performed using ESD; (c) Histopathological examination of the resected specimen showing intramucosal adenocarcinoma in adenomatous components (size: 110 × 70 mm), tumor‐free resection margins, and no evidence of lymphovascular invasion; (d) Surveillance colonoscopy after 3 months showed no recurrence of the ESD scar; (e) Surveillance colonoscopy after 51 months showed an elevated lesion on the ESD scar; (f) En bloc resection with ESD was performed for the recurrent lesion, which was pathologically diagnosed as an adenoma (HM0, VM0).

All patients with local recurrent lesions survived, with no further recurrences. The median follow‐up period was 94 months (range, 53–183 months) after initial ESD. Table 3 lists the clinicopathological characteristics of the seven local recurrent lesions. Histologically, the five primary lesions were Tis carcinomas, and two lesions were T1 carcinomas. Two lesions were resected using the piecemeal method. The time to recurrence was 4–102 months. Five lesions underwent additional endoscopic resections and two required surgery. Furthermore, three recurrent lesions in three patients were post‐R0 resection of ESD after a long period. The first case of local recurrence (Figure 3, Case No. 5 in Table 3) was a Tis carcinoma with histologically complete resection. In this lesion, no recurrence was suspected on surveillance colonoscopy at 4 and 16 months after ESD; however, a recurrent lesion pathologically diagnosed as an adenoma was detected at 38 months and cured by additional ESD (Case No. 5 in Table 4). The second case (Figure 4, Case No. 2 in Table 3) was tubular adenocarcinoma in an adenoma with histologically complete resection. No recurrence was suspected on surveillance colonoscopy at 3, 12, and 26 months after ESD; however, the recurrent lesion on the ESD scar was detected at 51 months (Case No. 2 in Table 4). Additional surgery was required in two cases. Of these cases, one was a recurrent lesion which was evidently a deep submucosal invasion, and the other was considered unsuitable for additional endoscopic treatment as the recurrent lesion was located on the ileocecal bulb and only a small section of it was visible.

**TABLE 3:** Characteristics of initial lesions that recurred after endoscopic submucosal dissection (ESD).

Characteristics of initial lesions										
Case no.	Age (years)	Sex	Location	Size (mm)	Morphology	Depth of invasion/	Resection method	Endoscopic curability	Curative resection	Fibrosis (F2)
**1**	81	M	D	40	Non‐polypoid (LST‐NG)	Sm (≥1000 μm)	Piecemeal	Non‐R0	Incomplete	−
**2**	73	M	S	110	Non‐polypoid (LST‐G)	M	En bloc	R0	Complete	−
**3**	84	M	B	40	Non‐polypoid (LST‐NG)	M	En bloc	Non‐R0	Incomplete	−
**4**	58	M	R	46	Non‐polypoid (LST‐G)	M	En bloc	R0	Complete	−
**5**	63	M	T	90	Non‐polypoid (LST‐G)	M	En bloc	R0	Complete	−
**6**	64	M	S	45	Polypoid (protruded type)	M	Piecemeal	Non‐R0	Incomplete	+
**7**	63	F	R	228	Non‐polypoid (LST‐G)	Sm (<1000 μm)	En bloc	Non‐R0	Incomplete	−

Abbreviations: B, Bauhin valve; D, descending colon; F, female; LST‐G, laterally spreading tumor‐granular; LST‐NG; laterally spreading tumor‐nongranular; M, male; R, rectum; S, sigmoid colon; T, transverse colon.

**TABLE 4:** Characteristics of local recurrent lesions after endoscopic submucosal dissection.

Characteristics of local recurrent lesions					
Case	Time to recurrence	Size	Morphology	Depth	Treatment method
No.	(months)	(mm)		of invasion	
**1**	4	6	Non‐polypoid (depressed type)	sm (≥1000 μm)	Surgery
**2**	51	12	Non‐polypoid (LST‐NG)	M	ESD
**3**	7	40	Non‐polypoid (LST‐NG)	M	Surgery
**4**	102	12	Polypoid (protruded type)	M	ESD
**5**	38	16	Non‐polypoid (LST‐NG)	M	ESD
**6**	11	8	Polypoid (protruded type)	M	EMR
**7**	5	26	Polypoid (protruded type)	M	ESD

Abbreviations: EMR, endoscopic mucosal resection; ESD, endoscopic submucosal dissection; LST‐NG, laterally spreading tumor‐nongranular.

## DISCUSSION

We demonstrated the long‐term clinical outcomes of ESD in patients with colorectal neoplasms in a large‐scale series with over 1500 lesions and a median follow‐up period of approximately 5 years. Risk factors for local recurrence were identified as follows: the size of the lesion ≥40 mm, piecemeal resection, non‐R0 resection, histologically incomplete resection, and severe fibrosis (F2). To our knowledge, this study is the first to examine risk factors for local recurrence after colorectal ESD as the primary endpoint.

One‐piece resection by ESD had solid long‐term clinical outcomes, with extremely low recurrence rates if histologically complete resection was achieved. The safety margin of the normal tissue from the border of the lesion, visibility of the cutting site, cauterization of the resection margin, and accurate pathological evaluation is generally considered to contribute to a low recurrence rate.^15^, ^16^ Previously, a multicenter prospective cohort study (JSCCR research project) showed that the local recurrence rate for colorectal neoplasms ≥20 mm after ESD was 1.4%, with piecemeal resection during ESD as a significant factor associated with local recurrence.^21^ The recurrence rate was relatively high, with a piecemeal resection rate of 5.0% and non‐R0 resection rate of 27.5 % (197/716), compared with the rates of our study—1.4% (21/1539) and 2.8% (43/1539), respectively. Unlike previous studies, the rate of piecemeal resection in our longer follow‐up study was low, and that of R0 resection was high.

In a systematic review of midterm outcomes of ESD for colorectal neoplasms, Repici et al.^15^ reported a 0.07% local recurrence rate in patients with R0 resection, with a median follow‐up period of approximately 2 years. Another systematic review of midterm outcomes by Patel et al.^16^ reported a 1% overall local recurrence rate over a median follow‐up period of approximately 2 years. These reviews suggest that long‐term follow‐up studies are required to determine the outcomes of colorectal ESD. In our study, the local recurrence rate was 0.5% (7/1539) within a median follow‐up period of 72 months. We found that four recurrent lesions had positive horizontal margins on initial ESD. These four lesions recurred within a year; therefore, early surveillance was required after non‐R0 resection. In contrast, the other three recurrent lesions after R0 resection occurred at successive periods of surveillance after the initial successful ESD, even beyond 3 years.

Among a variety of factors including clinical characteristics, and endoscopic and pathological features, the univariate analysis identified five factors as possible risk factors for local recurrence. Oka et al.^8^ reported that local recurrence after endoscopic resection of colorectal neoplasms is possibly due to residual tumors after the initial endoscopic resection. Therefore, it is important to detect minute amounts of residual tumor tissue that surround the resected ulcer or ulcer bed after ESD—even when en bloc resection has been achieved—and to carefully evaluate and confirm the pathological horizontal margin.^19^ Additionally, implantation of exfoliated malignant cells has been suggested as a possible mechanism for local recurrence in colorectal anastomoses,^32^, ^33^, ^34^ leading to local recurrence after R0 resections. A previous meta‐analysis has suggested that intraoperative rectal washout during rectal cancer surgery is recommended to prevent local recurrence.^35^ Similarly, intraluminal lavage after ESD is an effective method for achieving the clearance of exfoliated tumor cells.^36^ However, in the current study, genetic mutations between the primary lesion and the recurrent lesion were not investigated; therefore, further investigation is required.

The conditions of the ESD scars were recorded by pit‐pattern diagnosis using magnifying chromoendoscopy. There are no studies regarding the rate of negative biopsies from ESD scars; however, many published studies have demonstrated that pit‐pattern diagnosis reliably predicts histopathology.^37^, ^38^ In fact, pit‐pattern diagnosis using magnifying chromoendoscopy for colorectal neoplasms has 96% diagnostic accuracy.^39^ Therefore, we did not perform biopsies, even if a recurrent lesion was suspected, as it was considered sufficient to evaluate recurrence using only pit‐pattern diagnosis.^21^

Our study had several limitations. First, it was a single‐center retrospective study; however, we analyzed prospectively acquired data from consecutive patients. Second, not all patients undergoing ESD were followed up on; however, only 10.7% had no follow‐up, and the 89.3% rate of surveillance colonoscopy was relatively high, comparable to that of a recent prospective, multicenter, long‐term investigation after ESD (82.9%).^19^ Third, this study included patients that were treated since the introduction of ESD for colorectal neoplasms, albeit without improvements in the associated tools and devices. Fourth, multivariate analysis was not performed due to only seven local recurrence cases.

In conclusion, our study using long‐term surveillance colonoscopy after ESD identified five risk factors for local recurrence: the size of lesion ≥40 mm, piecemeal resection, non‐R0 resection, histologically incomplete resection, and severe fibrosis (F2). Patients with such factors should undergo careful surveillance colonoscopy.

## CONFLICT OF INTEREST STATEMENT

Masashi Misawa is an Associate Editor of Digestive Endoscopy. The other authors declare no conflict of interest.

## Data Availability

All data generated or analyzed during this study are included in this published article. OR The datasets generated during and/or analyzed during the current study are available from the corresponding author upon reasonable request.
